# Functional Characterization of Circulating Tumor Cells with a Prostate-Cancer-Specific Microfluidic Device

**DOI:** 10.1371/journal.pone.0035976

**Published:** 2012-04-27

**Authors:** Brian J. Kirby, Mona Jodari, Matthew S. Loftus, Gunjan Gakhar, Erica D. Pratt, Chantal Chanel-Vos, Jason P. Gleghorn, Steven M. Santana, He Liu, James P. Smith, Vicente N. Navarro, Scott T. Tagawa, Neil H. Bander, David M. Nanus, Paraskevi Giannakakou

**Affiliations:** 1 Sibley School of Mechanical and Aerospace Engineering, Cornell University, Ithaca, New York, United States of America; 2 Biomedical Engineering, Cornell University, Ithaca, New York, United States of America; 3 Division of Hematology and Medical Oncology, Department of Medicine, Weill Cornell Medical College and New York Presbyterian Hospital, New York, New York, United States of America; 4 Department of Urology, Weill Cornell Medical College and New York Presbyterian Hospital, New York, New York, United States of America; University of Kentucky College of Medicine, United States of America

## Abstract

Cancer metastasis accounts for the majority of cancer-related deaths owing to poor response to anticancer therapies. Molecular understanding of metastasis-associated drug resistance remains elusive due to the scarcity of available tumor tissue. Isolation of circulating tumor cells (CTCs) from the peripheral blood of patients has emerged as a valid alternative source of tumor tissue that can be subjected to molecular characterization. However, issues with low purity and sensitivity have impeded adoption to clinical practice. Here we report a novel method to capture and molecularly characterize CTCs isolated from castrate-resistant prostate cancer patients (CRPC) receiving taxane chemotherapy. We have developed a geometrically enhanced differential immunocapture (GEDI) microfluidic device that combines an anti-prostate specific membrane antigen (PSMA) antibody with a 3D geometry that captures CTCs while minimizing nonspecific leukocyte adhesion. Enumeration of GEDI-captured CTCs (defined as intact, nucleated PSMA+/CD45− cells) revealed a median of 54 cells per ml identified in CRPC patients versus 3 in healthy donors. Direct comparison with the commercially available CellSearch® revealed a 2–400 fold higher sensitivity achieved with the GEDI device. Confocal microscopy of patient-derived GEDI-captured CTCs identified the TMPRSS2:ERG fusion protein, while sequencing identified specific androgen receptor point mutation (T868A) in blood samples spiked with only 50 PC C4-2 cells. On-chip treatment of patient-derived CTCs with docetaxel and paclitaxel allowed monitoring of drug-target engagement by means of microtubule bundling. CTCs isolated from docetaxel-resistant CRPC patients did not show any evidence of drug activity. These measurements constitute the first functional assays of drug-target engagement in living circulating tumor cells and therefore have the potential to enable longitudinal monitoring of target response and inform the development of new anticancer agents.

## Introduction

The development of metastases in patients with solid tumor malignancies is believed to result from tumor cells entering the circulatory system and migrating to distant organs, where they extravasate and multiply [1]–[3]. Although circulating tumor cells (CTCs) are rare—as few as one cell per 100 million blood cells [3], [4], molecular and functional analyses of CTCs may provide a greater understanding of the biology of cancer metastases, help identify novel therapeutic targets, and enable clinical correlations to monitor patients undergoing treatment [5]. A variety of technologies has been developed to improve the detection and capture of CTCs from the peripheral blood. These include density gradient centrifugation, immunomagnetic bead separation using monoclonal antibodies targeting epithelial cell-surface antigens, cell sorting using flow cytometry, filtration based size separation [6] and microfluidic devices. Although advances in CTC capture have been made, the low frequency of CTCs in cancer patients, their heterogeneity, the lack of organ-specific capture approaches, and the plasticity of the CTC population has limited the ability to capture and track all CTCs [2], [6]. Currently, the epithelial cell-adhesion molecule (EpCAM), represents the antigen of choice for the majority of microfluidic devices that have been developed to capture circulating tumor cells [7]–[10].

However, accumulating evidence suggests that the expression of EpCAM during cancer progression and in particular during epithelial-to-mesenchymal transition has not been well characterized, raising concerns about the universality of this antigen for immunocapture systems [11], [12]. EpCAM has been reported to have oncogenic potential [13] and correlate with proliferation in cell lines [14]; however, it is downregulated during EMT [1], and EMT markers have been shown to be more important than epithelial markers e.g., cytokeratin in predicting cancer progression [15]. Thus, while EpCAM is clearly useful in identifying CTC populations in many cancers, the biases associated with EpCAM enrichment are currently unknown.

In addition to the uncertainties regarding surface antigens, the specificity of immunocapture from the blood is confounded by the non-specific adhesive properties of leukocytes on most antibody surfaces. Because of the presence of numerous leukocytes in blood at an approximately 10^4^–10^5^∶1 ratio with respect to the CTCs, immunospecific surfaces enrich CTCs but cannot isolate them from contaminating leukocytes entirely. Identifying CTCs requires additional steps and often involves staining with DAPI to ensure the presence of an intact nucleus and immunostaining to identify the presence of epithelial markers (i.e., cytokeratin) and the lack of the leukocytic marker CD45. Such immunostaining has identified a family of criteria that correlate CTC number with patient prognosis [16], but it requires that fixation and staining be central to CTC identification, as in the commercial CellSearch® system [17], [18]. Although enumeration of CTCs from patients with advanced prostate cancer receiving chemotherapy using the commercially available CTC capture system by CellSearch® has demonstrated utility as a prognostic indicator of patient survival [17], [18] and enumeration is currently being studied in a number of clinical trials, the presence of contaminating leukocytes impede the downstream utility of CTC capture devices, in that assays based on RNA or protein quantification are obfuscated by the need for fixation and material of leukocytic origin.

To facilitate high-efficiency capture of prostate CTCs, we have developed a prototype microfluidic device that employs an approach that we term geometrically enhanced differential immunocapture (GEDI). This device combines a geometry that reduces capture of contaminating leukocytes by generating size-dependent cell-wall collisions. We combine this geometric approach with a prostate-specific immunocapture surface using the J591 monoclonal antibody that recognizes the extracellular domain of prostate-specific membrane antigen (PSMA) [10]. PSMA is a cell surface peptidase highly expressed by malignant prostate epithelial cells. PSMA is an attractive target for prostate cancer CTC capture, as it is expressed on virtually all prostate cancer cells and expression increases following castration. We report here a detailed demonstration of the multiple utilities of the GEDI device, including a comparison of CTC enumeration with CellSearch®, the detection of a specific AR mutation from blood samples spiked with only 50 cells; the identification of the TMPRSS2-ERG fusion by immunostaining, and the *ex-vivo* assessment of CTC sensitivity to taxane-treatment using microtubule bundling as a marker of drug-target engagement.

## Materials and Methods

### Device Fabrication

All device fabrication was carried out at the Cornell NanoScale Science and Technology Facility (Ithaca, NY). Standard photolithography techniques were used to define array geometries on silicon wafers. The wafers were etched with an oxygen plasma deep reactive ion etcher (Uniaxis SLR770) to a depth of 100 µm, and cleaned using sulfuric acid and hydrogen peroxide prior to antibody surface functionalization. The J591 monoclonal antibody (manufactured by Lonza plc (Slough, England) for BZL Biologics, inc.) was immobilized on the device surfaces using MPTMS-GMBS-NeutrAvidin-biotin chemistry [10]. Polydimethylsiloxane (PDMS) sheets (5∶1 base∶curing agent), approximately 3 mm thick, were polymerized for 18 hrs at 60°C and trimmed to form covers for the GEDI device. A PDMS sheet was clamped to the top of the device with a custom jig to create closed channels populated with post arrays. Inlet and outlet holes were created with a biopsy punch, and 23-gauge metal tubes were inserted into the PDMS to connect inlet and outlets to external tubing. Devices were primed with a 50/50 isopropanol/water mixture, and then flushed with DI water and PBS before experiments.

### Sample Collection and Microfluidic Capture

Peripheral blood samples were collected in tubes containing sodium citrate anticoagulant (Becton-Dickinson) from healthy volunteers and patients with metastatic castrate-resistant prostate cancer under a clinical protocol entitled “Analysis of circulating tumor cells in prostate cancer. Predicting response to taxanes: a pilot study" which was approved by the Institutional Review Board (IRB) of Weill Cornell Medical College of Cornell University. Blood was obtained from patients or healthy donors following written informed consent, which was also approved by the IRB committee of Weill Cornell Medical College of Cornell University. As previously described [10], 1 ml of blood from each specimen was processed through the GEDI chip within 24 hr of blood draw by pushing the blood through the device at a volumetric flow rate of 1 ml/hr (Chemyx syringe pump).

### Cell staining and analysis

The cell lines used in these experiments as controls for staining or in spiked experiments are: the human leukemia cell line U937, and the human prostate cancer cell lines PC-3, LNCaP and C4-2. All cell lines were purchased from ATCC. Post-capture, cells were fixed on-chip with PHEMO fixative at 37°C (PHEMO buffer: PIPES acid, HEPES acid, EGTA disodium salt, Mg-Cl2-6H_2_0, 10%DMSO), gluteraldehyde, and 3.7%formaldehyde. Cells were then blocked (10% Normal Goat Serum – Jackson Immuno Research) and immunostained with FITC-conjugated humanized mAb J591 to detect PSMA expression. Monoclonal mouse anti-CD-45 (BD Biosciences) followed by AlexaFluor568 labeled goat anti-mouse secondary (Invitrogen) and mouse anti-EpCAM directly conjugated to AlexaFluor647 (Biolegend). For the detection of intracellular antigens, cells were permeabilized with 0.1% Triton X-100 (Sigma-Aldrich) in PBS and stained by use of rat anti-alpha tubulin (YL1/2, Millipore) and rabbit anti-ERG monoclonal antibody (clone EPR 3864; Epitomics, Burlingame, CA). The anti-ERG antibody was a generous gift from Dr. Mark Rubin (Weill Cornell Medical College, New York, NY). All primary antibodies were incubated for 1 hour at room temperature; secondary antibodies were stained at room temperature for 30 minutes. DAPI was used for DNA counterstaining. GEDI devices were mounted to coverslips with Mowiol and stored at −20°C before analysis.

### CTC enumeration

Blinded CTC enumeration following antibody labeling was performed by use of a Zeiss LSM-700 point scanning confocal microscope, equipped with 405-, 488-, 555-, and 632-nm laser lines. All PSMA+/CD45− nucleated cells were identified as CTCs. Initial validation of CTC enumeration was accomplished by two independent, blinded testers. Positive and negative controls for antibody performance and staining were included in each experiment: U937 human leukemia cells (CD45+/PSMA−/EpCAM−), and the human prostate cancer cell lines (C4-2 and LNCaP: PSMA+/CD45−/EpCAM+ and PC-3 (PSMA−/Cd45−/EpCAM dim). Individual *z*-stacks were acquired using 100X/NA 1.46 and 63X/NA 1.3 Plan-Apo Zeiss objectives controlled by Zen software (Zeiss) and presented as maximum intensity projections.

### RNA extraction

Following cell capture, the GEDI device was rinsed by flowing PBS for 30 min at a rate of 1 ml/hr. Cells were lysed with 700 µl of RLT Lysis buffer supplemented with 1% β-mercaptoethanol at a flow rate of 15 ml/hr. The lysate was collected, and RNA was extracted using the QiagenRNEasy Micro Plus Kit (Qiagen Inc, Valencia, CA) according to the manufacturer's instructions.

### Ex-vivo Drug treatments and Analysis

For *ex vivo* drug treatment experiments, the blood sample from each patient was divided and 1 ml was flown to each of three GEDI devices simultaneously. After CTC capture and subsequent PBS wash, each GEDI microdevice was gently placed in a culture dish with RPMI-1640 media containing 2% serum and supplemented with either 0.1% DMSO control or paclitaxel at concentrations of 100 nM or 1 µM and incubated at 37°C for 24 hr. At the end of treatment, the GEDI-captured cells were fixed with PHEMO buffer and processed for multiplex confocal microscopy following immunostaining with different cell surface and cytoplasmic antibodies as indicated. All CTCs (PSMA+/CD45−/DAPI+) were assessed for the presence of microtubule bundles as evidence of effective drug-target engagement. The percent of CTCs with evidence of microtubule bundling was calculated. In all samples analyzed, bundling was clearly apparent by the distinct shape, width, orientation and increased fluorescence intensity of microtubule bundles as compared with microtubules from untreated cells. In addition, we used DAPI counterstain to assess the presence of mitotic or apoptotic nuclei following drug treatment.

### Statistical Analysis

Statistical analysis was performed to compare the mean CTC counts obtained from CRPC patients and healthy donors. We used a non-parametric (Wilcoxon signed-rank) analysis, as CTC counts did not exhibit normal distribution. Statistical significance was defined with α = 0.05.

## Results

**Figure 1:**
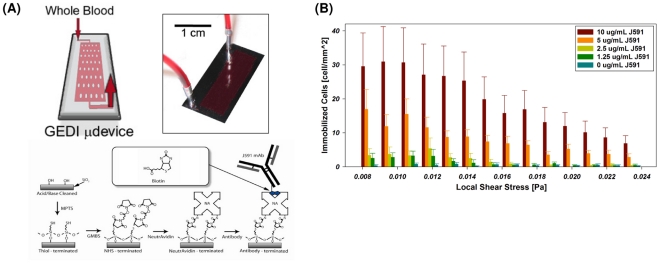
GEDI microfluidic device design. (A) GEDI device overview. Clockwise from upper left: schematic of blood flow through device, image of silicon device with silicone gasket, surface functionalization scheme. (B) Cell capture performance as a function of shear stress and antibody concentration. Titration curves for the anti-PSMA J591 antibody in a standardized geometry indicate optimal antibody concentration for cell capture.

To characterize the performance of the device (Figure 1A), we determined cell capture rates with PSMA-positive cancer cells. We determined cell capture as a function of varying mAb J591 concentrations (1.5–20 µg/ml) using shear stress magnitudes representative of those experienced by the functionalized surfaces of the device (0.08–0.24 Pa). These experiments revealed a dose-dependent increase in cell capture up to mAb concentration of 10 µg/ml, which was used for all subsequent experiments (Figure 1B).

**Figure 2:**
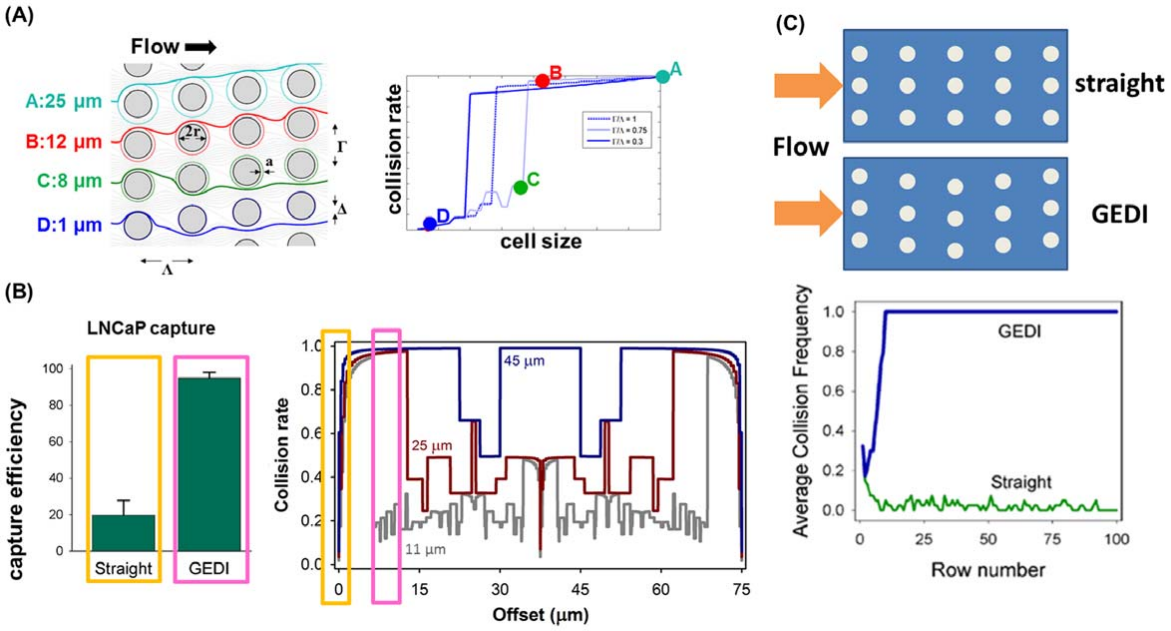
GEDI microfluidic device-results in cell lines recapitulate simulations. (A) Left: Top view of microfluidic obstacle array with array geometric parameters. Δ = obstacle offset. Λ = obstacle spacing in the direction of bulk flow. Γ = obstacle spacing in the direction orthogonal to bulk flow. 2*r* = obstacle diameter. Streamlines (gray) denote fluid flow. Pathlines (various colors) denote trajectories of cells of different diameters. Obstacle array spacing and orientation parameters are also defined. Right: The rate of cell-wall collisions for cells traveling through the array is a strong function of the offset parameter of the array; the GEDI design methodology implies use of an offset parameter that leads to size-dependent collision rates. The results predicted for the flow through the geometry at left are shown at right by the solid line; the four specific cell sizes lead to results denoted by the four colored dots on this graph. Other geometric arrangements lead to different results, shown at right in the dotted and dashed lines. (B) Straight arrays or arrays with small offsets (gold boxes) lead to lower capture efficiency (left) and size independence (right). Carefully chosen offsets (magenta boxes) lead to high capture rates (left) and size dependence (right). Capture rates at left compare GEDI (7-µm offset) and straight (no offset) geometry performance as measured by LNCaP capture efficiency on J591-functionalized devices. Rates at right describe simulated collision rates in these geometries. Both experimental results have the same surface-area-to-volume ratio. (C) Devices with the same surface area to volume ratio give vastly different results: straight arrays lead to collisions that decrease as the blood travels through the device; GEDI arrays lead to collisions that increase with travel through the device.

Although the J591 antibody is specific for PSMA-expressing cells, non-specific leukocyte adhesion has been a major problem for all blood-based immunocapture techniques. To minimize leukocyte adhesion, we conducted a parametric study to characterize collision rate (CpR; collision per row) as a function of cell size and obstacle offset. Collision rates from a subset of these offsets exhibit a sharp cutoff according to cell size, as shown in Figure 2A; hence we selected an obstacle offset (7 µm) that generates a sharp cutoff at the cell diameter of 14 µm. The physics describing this cutoff is best illustrated by the size-dependent cell pathlines (Figure 2A), which show how large cells experience repeated collision whereas small cells separate from the obstacles and escape capture. We then tested this hypothesis by measuring capture of LNCaP prostate cancer cells (Figure 2B). In this experiment, spiked LNCaP cells were flown into J591-functionalized devices that had a 7 µm offset (GEDI) *versus* those that had no offset (straight). Although these two devices have the same surface-area-to-volume ratio, the GEDI geometry greatly increased cell capture efficiency, as measured by captured and enumerated cells normalized by input cell counts.

Because of the dependence of cell trajectory on cell diameter, collision rates are a complicated function of both cell diameter and array parameters such as row offsets. The collision rate per row (CpR) is a strong function of the row offset, exhibiting discontinuities, size dependence, and startup effects related to the finite array size (Figure 2C). The dramatic difference between performance of different designs is caused by the deflection of particles—in poorly-chosen geometries, the deflection causes cells to deflect onto streamlines that do not come into proximity with later obstacles, whereas in well-chosen geometries, the deflection causes cells to deflect onto streamlines that do come into proximity with later obstacles. Thus the collision rate increases as the cells proceed through the device for the GEDI design, and decreases for poorly chosen designs such as straight arrays (Figure 2C). This cutoff allows the user to identify a cutoff between hematocytes (<14 µm) and the cell population that will experience maximum collisions (>15 µm).

### Cell capture, imaging, and enumeration of CTCs from metastatic prostate cancer patients

**Figure 3:**
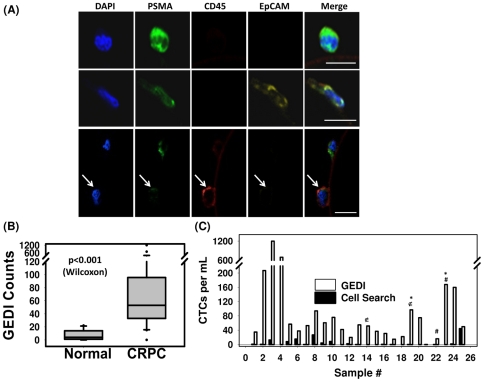
CTC enumeration using the GEDI and comparison to capture by Cell Search. (A) Representative images of circulating tumor cells captured with the GEDI device from 1 mL of blood from prostate cancer patients. CTCs are imaged on the device and are identified following immunostaining with DAPI, PSMA, CD45, and EpCAM. Intact, nucleated cells that are PSMA+/CD45− are identified as CTCs. Leucocytes are identified as DAPI+/PSMA−/CD45+ (bottom row, arrow). Note the heterogeneity of EpCAM expression in the PSMA+ cell population (top and bottom rows, EpCAM-; middle row, EpCAM+). Scale bar: 10 µm. (B) Disease-specific GEDI capture of CTCs. CTC enumeration (CTCs/ml) was performed using blood from healthy donors (median = 3) and CRPC patients (median = 54). (p<.001; Wilcoxon) (C) Comparison of CTC enumeration between GEDI-based- and CellSearch®-based capture. This comparison was performed using same-day blood draws from 25 individual CRPC patients. * indicates that CellSearch-based enumeration was performed 1 week before GEDI-based enumeration; ∉ indicates same patient whose blood was drawn on two separate time points three months apart (blood draw no 14 occurred 3 months after blood draw no 19); # indicates same patient whose blood was drawn on two separate time points 1 year apart (blood draw no 22 occurred 1 year before blood draw no 23).

Next, we used the GEDI device to capture and characterize circulating tumor cells (CTCs) from the blood of patients with metastatic CRPC. One ml of peripheral blood was flown through the device, captured cells were fixed and immunostained for PSMA, CD45, EpCAM and DAPI, and the captured cells were analyzed by confocal microscopy. CTCs were defined as intact, nucleated, PSMA+/CD45− cells. Representative examples of CTCs and leucocytes are shown in Figure 3A. Interestingly, PSMA+ cells had variable EpCAM staining, ranging from highly-positive to weak to negative in terms of EpCAM fluorescent intensity. In a subset of patients, we quantitated the percent of PSMA-captured CTCs that were EpCAM positive. We observed that 40–70% of GEDI-captured CTCs were positive for both markers, with the median being 60% (data not shown). Controls for antibody performance were included with every experiment using two prostate cancer cell lines expressing different levels of PSMA and EpCAM (C4-2:PSMA+/EpCAM+/CD45− and PC3: PSMA−/EpCAM−/CD45−) and the CD45+ leukemia cell line U937 (Figure S1A).

We processed blood obtained from 10 healthy donors (controls) and 30 patients with metastatic CRPC using the GEDI device. The median number of CTCs/ml detected was 3 (range 0 to 22) and 54 (range 0 to 1200), respectively (p<0.001; Figure 3B). Next, we performed a direct comparison of CTC capture and enumeration by comparing the GEDI microdevice with the FDA-approved EpCAM-based CellSearch® CTC Test on same-day blood draws from 25 CRPC patients (Figure 3C). We detected a 2 to 400-fold increase in the number of CTCs/ml reported with the GEDI microdevice relative to the CellSearch ® CTC Test (Figure 3C; *p*<.0001, calculated with Wilcoxon test), and a weak correlation (*r* = 0.44; outliers removed with Cook's distance restriction) between GEDI CTC counts and CellSearch® (Figure S2).

### Molecular characterization of captured cells: Detection of a single point mutation in the androgen receptor and expression of the TMPRSS2-ERG fusion in spiked cells and CTCs

**Figure 4:**
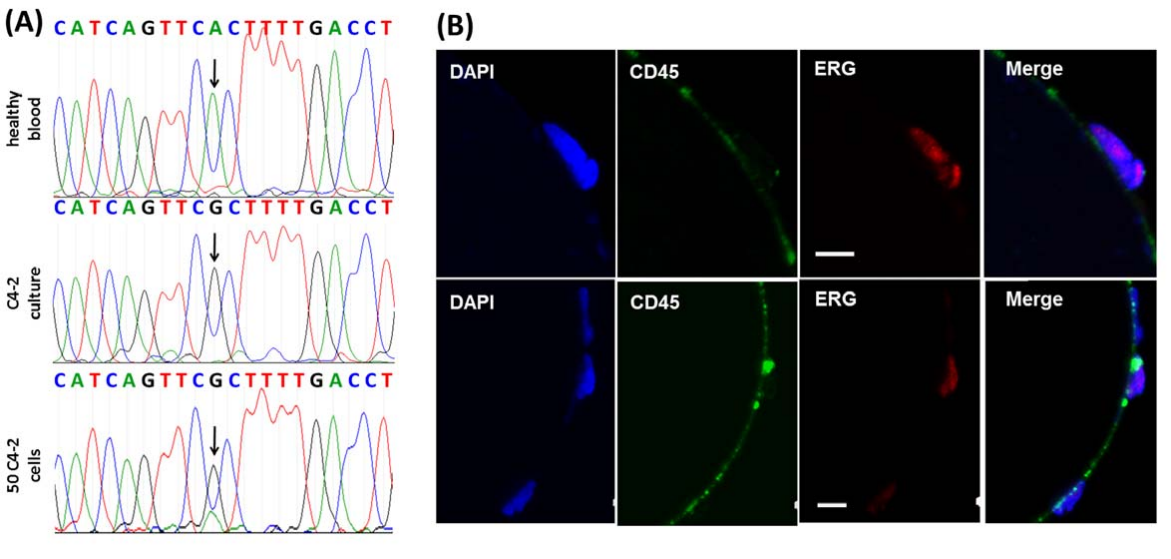
Functional characterization and detection of genetic alterations in GEDI-captured cells. (A) Captured cells exhibit high purity, enabling the identification of single point mutations. The T868A (ACT-GCT; Thr-Ala) AR single-point mutation is detected from RNA extracted from 50 C4-2 cells spiked into 1 ml of healthy-donor blood and captured by the GEDI device (third row, arrow). Sequencing results from 1 ml blood from the same healthy donor (top row) or from 50 C4-2 cells in culture (middle row) are also depicted. (B) The TMPRSS2:ERG fusion protein is detected in GEDI-captured CTCs from a CRPC patient. PSMA-captured CTCs were stained on the device with an anti-ERG antibody. Representative examples of three PSMA+/CD45− CTCs are shown, two of which are positive for ERG. Scale bars: 10 microns.

To assess whether we could molecularly characterize the GEDI-captured CTCs, we performed proof-of-principle experiments in which prostate cancer cells were spiked into 1 ml of blood from a healthy donor, captured by the device and analyzed for the presence of androgen receptor (AR) point mutations or expression of TMPRSS2:ERG gene fusion protein. To detect the T868A (ACT-GCT) single point mutation in the AR ligand-binding domain [19], 50 C4-2 cells were added into 1 ml of healthy donor blood flown through the GEDI device, and RNA was extracted by direct lysis on the device followed by cDNA sequencing (Figure 4A). In parallel, sequencing was performed on RNA extracted from 1 ml of the same donor blood processed similarly (negative control) and on RNA extracted from 50 C4-2 cells (positive control). As expected, the T868A mutation was clearly detected in the C4-2 cells (Figure 4A, top and middle panel) but absent from the negative control. The point mutation was also detected in the spiked blood sample, with the mutant peak (A) accounting for 70% of the nucleotide present at this position and the wild-type (T) for 30%. This result is consistent with the previously reported cell capture purity rate of 68% obtained with fluorescently labeled prostate cancer cells spiked into 1 ml peripheral blood from a healthy donor and flown through the GEDI microdevice [10].

In addition, the presence of the TMPRSS2:ERG fusion protein could be detected by immunostaining with the rabbit monoclonal anti-ERG antibody [20] in fusion-positive vCaP prostate cancer cells captured by the device and analyzed by multiplex confocal microscopy (Figure S3). Similar analysis on GEDI-captured CTCs from a CRPC patient revealed the presence of both ERG positive and ERG negative cells, while CD45+ leucocytes were negative for ERG protein (Figure S3, panel C), indicating the specificity of ERG staining for prostate cancer-derived cells.

### Functional assays: tubulin bundling upon exposure to taxanes

Given the high purity and viability of the GEDI-captured CTCs, we next tested the hypothesis that CTC chemosensitivity to taxanes following *ex vivo* treatment on the GEDI microdevice could predict a patient's clinical response to therapy. Taxanes (paclitaxel, docetaxel and cabazitaxel), which are commonly used to treat CRPC patients [21]–[23] act by stabilizing microtubule polymers and inducing the formation of microtubule bundles. Microtubule bundling is readily detectable by immunofluorescence staining, due to their increased fluorescence intensity, distinct shape and cytoplasmic organization when viewed in maximum signal projection [24]. Microtubule bundling is the first event induced by taxane treatment, resulting in downstream mitotic arrest and apoptotic cell death, and is therefore an appropriate marker of efficient taxane drug-target engagement.

**Figure 5:**
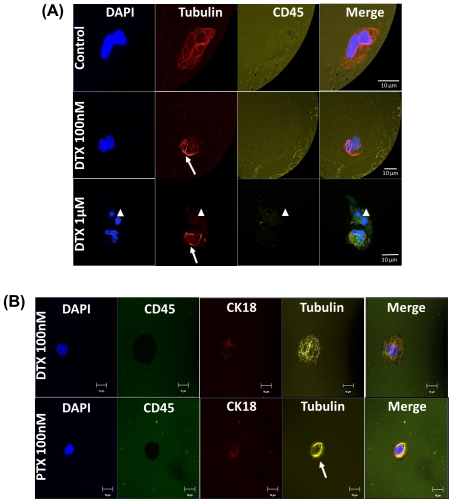
On-chip assessment of effective drug-target engagement on viable GEDI-captured CTCs. Tubulin response to taxane treatment can be assessed in GEDI-captured cells. (A) C4-2 prostate cancer cells were spiked at 200 cells/ml into healthy-donor whole blood. One ml of spiked blood was then flowed in each of three GEDI devices. Captured cells were incubated on each device at 37°C for 24 hr with either DMSO control (upper panel) or DTX 100 nM (middle panel) or 1 µM (lower panel). Following drug treatment, cells were fixed and processed for immunofluorescence staining using antibodies against tubulin and CD45. DAPI was used as a DNA counterstain to evaluate nuclear integrity. Note the fine and intricate microtubule network in the DMSO control (top panel) and the distinct microtubule bundles in the DTX treated devices (arrows, middle and bottom panels). Apoptotic nuclei were observed at higher DTX concentrations (arrowhead, bottom panel). (B) GEDI-captured CTCs from the blood of a CRPC patient were treated *ex vivo* on the GEDI device with 100 nM DTX (top panel) or the addition of 100 nM PTX (bottom panel) at 37°C for 24 hr. Following drug treatment the PSMA-captured cells were fixed and processed for immunofluorescence staining as in (A) with the addition of cytokeratin-18 as an alternative epithelial marker. In this patient, the presence of an unperturbed microtubule network following DTX treatment (top panel) indicates lack of efficient drug-target engagement. In contrast, addition of PTX resulted in microtubule bundling (bottom panel).

To first determine the optimal concentration and duration of *ex vivo* on-chip treatment of patient-derived CTCs, we spiked 200 C4-2 cells to 1 ml of blood from a healthy donor, processed the sample in the GEDI device, and then incubated the captured cells on the device for 24 hr in media containing 100 nM or 1 µM of docetaxel (DTX) at 37°C. Docetaxel treatment with 100 nM resulted in distinct microtubule bundles in captured C4-2 cells (Figure 5A, middle panel arrows), in contrast to the fine and intricate microtubule network of the untreated GEDI-captured cells (Figure 5A, upper panel). Treatment with 1 µM DTX resulted in robust microtubule bundling and induction of apoptotic events (Figure 5A, lower panel arrowheads). Apoptotic events were determined by the presence of bright and fragmented nuclei accompanied by loss of tubulin staining. Similar results were obtained with 48 hr, on-chip, *ex vivo* treatment (data not shown). These results, together with the fact that the AUC of serum docetaxel concentration for patients administered 55–100 mg/m^2^ of docetaxel is approximately equal to 1.5–5 hrs mg/l [25], led us to select a 24 hr treatment with 100 nM of a taxane (1.92 hours mg/l) in order to evaluate the *ex vivo* response of patient-derived CTCs.

A number of assays have been performed to show the potential for functional assay in the described device. In these cases cells captured in the device were treated *ex vivo* with DTX and/or PTX for 24 hr (Figures 5B and S3). These highlight the ability to perform functional assays on chip and assay drug-target engagement in patients in the context of their clinical response. Non-response, as indicated by a lack of evidence of microtubule bundling or apoptotic nuclei following *ex vivo* DTX treatment, could be observed in some patients. Figure S4C shows a patient that was non-responsive by the microtubule assay, consistent with this patient's lack of a clinical response using established RECIST and PSAWG2 criteria. This response was often heterogeneous within the captured cell population; Fig. S4D shows a patient with heterogeneous response within the PSMA+ CTC population, with only 45% of CTCs displaying clear evidence of microtubule bundling (whereas nearly 100% of the leucocytes present on the chip showed microtubule bundling). Interestingly, cell response was often specific to the taxane used. As an example of this, Figure 5B (top panel) shows a sample in which *ex vivo* DTX had no effect on the microtubule cytoskeleton of captured CTCs, whereas isolated CTCs from the same patient (DTX progressor) treated with PTX demonstrated clear evidence of microtubule bundling (Figure 5B, bottom panel) as well as signs of aberrant mitotic arrest (Figure S4A). Figure S4B shows an example of a patient (DTX progressor on cabazitaxel) whose CTCs exhibited apoptotic nuclei with PTX but not with DTX.

## Discussion

The overwhelming majority of cancer-related deaths result from the development of hematogenously disseminated metastasis. A central challenge in the development of effective treatments for metastatic disease is the lack of understanding of the molecular evolution of the tumor from the localized to the metastatic state. This is attributable to the lack of readily available tumor tissue that can be obtained from patients over the course of their disease and analyzed for molecular signatures and functional response. The identification of circulating tumor cells in the peripheral blood of metastatic patients and the development of specialized CTC-capture technologies has provided, for the first time, investigators, with tumor tissue in the form of a “liquid biopsy" that can be used to study the process of metastasis and aid in the development of effective therapies [26].

In the past decade, there has been an exponential increase in the number of assays developed to isolate CTCs from metastatic cancer patients. Among those, the only FDA-approved and most widely used method is the CellSearch® technology, which relies on immunocapture of CTCs using antibodies against the cell surface epithelial cell adhesion molecule (EpCAM). EpCAM-based enrichment followed by staining for cytokeratin and CD45 has been shown to enumerate CTCs and correlate with patient prognosis [16]. Several studies using this technology have shown that increased CTC numbers (as determined by CellSearch®) are negatively correlated with prognosis in patients with metastatic breast [27], colorectal [28] and prostate cancer [29]. However, the clinical utility of monitoring CTC numbers remains controversial [30] and CellSearch® studies have failed to impact clinical care, as CellSearch® has to date not created an indication for which treatment strategy has changed [31]; the recent ASCO guidelines do not recommend the clinical use of the CellSearch® assay until further validation confirms its clinical value [6], [32].

Our work is motivated by the need in the field to move beyond CTC enumeration to molecular characterization of CTCs. A major challenge that remains, though, is the capture of CTCs with high enough purity to allow accurate molecular characterization and their viability to enable functional assays and biomarker discovery for clinical application [31], [33], [34]. The presented microdevice is the only microfluidic device reported to date that utilizes an antibody other than EpCAM, namely PSMA, for CTC immunocapture (although other antibodies have been used for cell line capture). The prostate-specific membrane antigen (PSMA; folate hydrolase 1; glutamate carboxypeptidase II) is a type II (i.e. extracellular C-terminus) transmembrane metallopeptidase with an extracellular domain accessible to antibodies, and that has been immunolabeled for imaging or targeted therapy [35]. PSMA is expressed by virtually all prostate cancer primary tumors [36]–[39], and overexpressed in higher grade cancers, castrate-resistant cancer and metastatic disease.

In contrast to EpCAM, which is thought to be downregulated as neoplastic cells begin to exhibit a mesenchymal phenotype [1], PSMA is largely specific to prostate cells while its expression increases progressively in higher grade PC, metastatic disease and CRPC [40]. Importantly, PSMA expression is upregulated following androgen-deprivation therapy [41] and as such it is highly expressed in metastatic CRPC patients. Interestingly, PSMA expression in other cell types, for example the vascular endothelium (but not the primary epithelial tumor) of other tumors [42]–[44] does not correlate with disease progression [44]. Finally, a recent review highlighting 10 years of clinical experience using anti-PSMA mAb J591 radioimmunotherapy, showed that 86.6% of subjects across four phase I and phase II studies had positive PSMA scans of metastatic sites of disease on planar imaging [45]. Because of these characteristics, PSMA is an ideal surface antigen for identifying cancer cells of prostate origin.

In our work, we have defined CTCs as large, nucleated cells, PSMA+/CD45−. We have focused our enumeration and analysis only on intact cells that fulfill the CTC criteria and have ignored cell fragments, non-nucleated cells that otherwise would qualify as CTCs, despite a recent report suggesting that enumeration of such events can impact the prognostic value of the CTC assay [16]. Since the clinical correlates associated with PSMA-enriched circulating tumor cells are not yet established, we chose to apply more stringent criteria in our current CTC definition.

The results presented in Figure 3 illustrate both that the EpCAM expression level of captured PSMA+/DAPI+/CD45− cells is variable and that the correlation between GEDI PSMA+ capture and CellSearch® EpCAM+ capture is only weak. The two capture methodologies clearly both correlate with the disease state, but the population captured by the GEDI device is different from that captured by CellSearch®, presumably owing to different expression levels of EpCAM and PSMA in the CTC population. CellSearch®-identified CTCs are most commonly detected in metastatic prostate cancer and at higher numbers than breast and colorectal cancer [29]. Yet, our data show a significantly higher CTC detection using the GEDI microfluidic device compared with CellSearch®. This finding highlights the enhanced sensitivity of CTC detection in prostate cancer using the GEDI microdevice. Other microdevices have also reported increased sensitivity and detection levels in microfluidic geometries with anti-EpCAM capture relative to CellSearch® [7]–[9], so these data alone do not indicate that anti-PSMA capture leads to higher cell counts than anti-EpCAM capture. However, because CTCs expressing low EpCAM levels are frequently missed using CellSearch® [46], it is likely that anti-PSMA capture maybe be a more consistent methodology for CTC isolation in metastatic prostate cancer patients. To further investigate the distribution of the two antigens in CRPC isolated CTCs, in an unbiased manner (i.e. no antibody-specific CTC capture) we isolated CRPC-patient CTCs by performing immuno-magnetic CD45 depletion of Ficoll-isolated peripheral blood mononuclear cells. These immunodepleted cells were subsequently labeled with antibodies against PSMA (J591), EpCAM and CD45. In this assay, CTCs were classified as nucleated CD45− cells that were positive for either PSMA, EpCAM or both. We analyzed a total of eleven patient samples and have identified CTCs in seven patients. Our results revealed over 80% of dual PSMA+/EpCAM+ CTCs in six of the seven patients (Figure S5A and B). Interestingly, using this technique, we also found that EpCAM staining intensity was variable which is consistent with the data obtained using the GEDI-microdevice (Figure 3).

Taken together these data indicate that an important contributing factor to the enhanced sensitivity of the GEDI is that our microdevice is designed to optimize CTC capture and minimize leukocyte capture; it does this by inducing a fluid flow that generates size-dependent trajectories of cells that lead to size-dependent collision rates. Spatial separation of cells based on size alone is of limited use in CTC capture from blood—although CTCs tend to be larger on average that hematological cells, the sizes of cells and cell fragments of epithelial origin has a broad distribution, and size is much less specific to the CTC phenotype than surface markers such as EpCAM, PSMA, or EGFR. However, when a surface antibody is present, size-dependent particle trajectories enable the captured cell population to be biased to reject nonspecific leukocyte adhesion. The surface collision rate and capture rate (Figure 2) can be made size-specific, enhancing the receiver-operator characteristic of the rare cell capture. By capturing CTCs at high efficiency and purity, functional and molecular assays, exemplified by the ERG and SNP measurements in Figure 4 and the tubulin measurements in Figure 5 can be made applicable in a clinical setting.

ERG gene rearrangements are reported to occur only in neoplastic and preneoplastic tissue and not in normal tissue [47], [48]. Our findings suggest that the CTCs we detect are malignant in origin and heterogeneous in nature, as observed in previous studies of the presence of ERG gene rearrangement in CTCs using FISH [9], [49]. The advantage of the use of an antibody-based detection method for ERG is that is allows multiplexing for different cell markers that can further characterize tumor cell heterogeneity. Although it is well established that in primary PC there is heterogeneity in terms of ERG rearrangement, data is limited on ERG heterogeneity within a metastatic patient or potential changes in the ETS rearrangement event over the course of therapy, similar to HER-2 gene amplification that is enhanced as breast cancer progresses [50]. In addition, the impact of ERG on taxane sensitivity is not known, or potential enrichment of ERG-positive CTCs as a patient becomes refractory to taxane treatment. These are important clinical questions that can be answered prospectively in clinical trials that incorporate the GEDI-based biomarker studies.

A major challenge in the clinical management of CRPC is that currently there is no biomarker that predicts clinical efficacy of chemotherapy. The taxanes represent the only class of cancer chemotherapeutics demonstrated to prolong survival in CRPC [22], [23]. Although clinically defined patient groups inform the statistical efficacy of microtubule-targeting agents, no predictive markers are in use to direct microtubule-targeted therapy on an individual-patient basis. Despite their clinical success, treatment efficacy can be transient and the development of clinical taxane resistance is the major cause of cancer-related death. As there are two FDA-approved taxanes for CRPC, the important clinical question is to understand why patients who fail treatment with one taxane respond to another, and how clinicians can anticipate progression and proactively switch treatment.

At the cellular level, taxanes bind and stabilize microtubules, compromising their function during interphase [51] as well as mitosis [52], ultimately inducing cell death. However, these effects are variable between patients and may be variable within the metastases and circulating tumor cell population of an individual patient. Historically, investigators have searched for surrogate markers predictive of taxane clinical activity, albeit without success. Attempts to correlate drug-induced microtubule stabilization by quantitating the percent of patient derived PBMCs with microtubule bundles and correlating these results with clinical response to therapy bore no correlation [53]. This result may not be surprising given that PBMC biology is entirely distinct from that of tumor tissue. In fact, our results do not indicate any relation between tubulin bundling in leucocytes and clinical response to taxane chemotherapy (Figure S4D). On the other hand, absence of taxane-induced microtubule bundling following *ex vivo* treatment of GEDI-captured CTCs trended with clinical progression on the same taxane (Figure 5). Interestingly, our results show differential CTC response to docetaxel versus paclitaxel, which recapitulates the known clinical observation for lack of cross-resistance between the three taxanes used for CRPC treatment. Furthermore, these observations generate the hypothesis that *ex vivo* drug-target engagement of the GEDI-captured CTCs combined with the use of other relevant biomarkers, such as inhibition of androgen receptor nuclear accumulation downstream of taxane-induced microtubule stabilization, [54] may predict clinical response and help identify a subset of patients more likely to benefit from treatment with a specific taxane.

The presented work demonstrates the first functional assay of a microtubule-targeting agent on living circulating tumor cells microfluidically extracted from patient blood. This work highlights the potential for tailoring of chemotherapy by real-time monitoring of drug-target engagement in CTCs. Further, the demonstrated ability to identify genetic mutations and fusions in rare cell populations points to the potential for identifying mechanisms underlying clinical response and resistance.

## Supporting Information

Figure S1 **Multiplex immunostaining for specific cell surface markers.** Different cell lines were used as controls for antibody staining for PSMA, CD45, and EpCAM, as follows: the C4-2 prostate cancer cells are PSMA+/EpCAM+/CD45− while the PC3 prostate cancer cells are PSMA−/EpCAM−/CD45−. The U937 leukemic cell line was used as a positive control for the leukocyte marker CD45. DAPI was used to stain the DNA.
(TIFF)

Figure S2 **GEDI-CellSearch correlation.** Correlation between the number of CTCs detected by the CellSearch® system vs. the GEDI system from same day blood draws. A correlation coefficient of *r* = 0.44 (outliers were removed with Cook's distance restriction) was determined. Hashtag and asterisk denote two pairs of data each taken on the same patient at two longitudinal time points. *r* is not changed significantly by inclusion or rejection of these points.
(TIFF)

Figure S3 **TMPRSS2:ERG detection by immunofluorescence on GEDI-captured cells.** (A) The performance of the ERG antibody staining was tested in TMPRSS2:ERG fusion-positive (vCaP) and fusion-negative (C4-2) prostate cancer cell lines. Representative images acquired by confocal microscopy are displayed. Note the nuclear ERG staining in fusion-positive vCaP cells. (B) Two hundred vCaP cells were spiked in 1 ml of healthy-donor blood, flown through the GEDI device and processed for ERG immunofluorescence labeling. Nuclear ERG staining was detected in the GEDI-captured vCaP cells, identified as PSMA+/DAPI+/CD45− cells. (C) Representative example of ERG-negative/CD45+ leucocytes identified in the blood from a CRPC patient processed by the GEDI device as in Figure 4B.
(TIFF)

Figure S4 **Additional examples of on-chip assessment of effective drug-target engagement from different CRPC patients: taxane-induced microtubule bundling and mitotic defects as evidence of drug-target engagement in GEDI-captured CTCs.** (A) GEDI-captured CTCs from the same patient as in Figure 5B. PTX-induced prometaphase arrest of GEDI-captured CTCs provides additional evidence of effective drug-target engagement. (B) GEDI-captured CTCs from patient 3 following *ex vivo* on-chip treatment with 100 nM DTX do not show any evidence of microtubule response (bundling) to drug treatment. (C) GEDI-captured CTCs from patient 2 display microtubule bundling (arrow) following *ex vivo* on-chip treatment with 100 nM or 1 µM PTX. (D) GEDI-captured CTCs from patient 4 following *ex vivo* on-chip treatment with 50 nM PTX show heterogeneous response to drug treatment. Note, distinct microtubule bundling in a PSMA+ CTC (middle panel, barbed arrow) and no detectable microtubule network in another PSMA+ CTC from the same patient (bottom panel, standard arrow). The adjacent leucocyte (PSMA−) shows clear microtubule bundling in response to PTX treatment.
(TIFF)

Figure S5 **PSMA and EpCAM expression in CRPC patient CTCs isolated using CD45− immunodepletion.** (A) Table showing the percentage of both PSMA and EpCAM positive CTCs from CRPC patients obtained using CD45-immunodepletion. (B) Representative images of CTCs isolated from 2 prostate cancer patients, stained for PSMA (Green) and EpCAM (Red) and analyzed by point scanning confocal microscopy. Scale Bar = 10 µm. The yellow arrows point to PSMA+/EpCAM dim staining. Notice the variability in EpCAM fluorescence intensity within each sample.
(TIF)
