# Correction: Functional Characterization of Circulating Tumor Cells with a Prostate-Cancer-Specific Microfluidic Device

**DOI:** 10.1371/annotation/9d3d22ed-dc5a-4484-9254-1584864f4aac

**Published:** 2012-07-09

**Authors:** Brian J. Kirby, Mona Jodari, Matthew S. Loftus, Gunjan Gakhar, Erica D. Pratt, Chantal Chanel-Vos, Jason P. Gleghorn, Steven M. Santana, He Liu, James P. Smith, Vicente N. Navarro, Scott T. Tagawa, Neil H. Bander, David M. Nanus, Paraskevi Giannakakou

There were errors in the Author Contributions. The correct contributions are:

Conceived and designed the experiments: BK DN PG JG EP SS JS.

Performed the experiments: EP SS JS ML CCV MJ GG VN.

Analyzed the data: BK DN ST PG JG EP CCV SS JS ML MJ GG.

Contributed reagents/materials/analysis tools: SS EP NB HL.

Wrote the paper: BK DN PG. 

