# Supplementary figures and images for: Functional Characterization of Circulating Tumor Cells with a Prostate-Cancer-Specific Microfluidic Device
Source: PLoS One. 2012 Apr 27;7(4):e35976. doi: 10.1371/journal.pone.0035976 (PMC3338784; doi:10.1371/journal.pone.0035976)

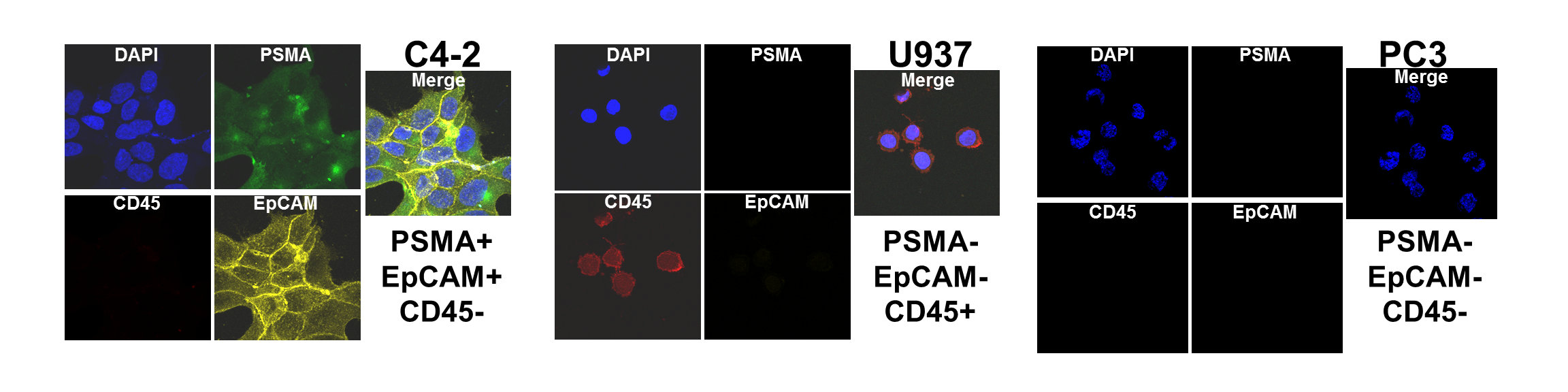

Supplement: Figure S1 — Multiplex immunostaining for specific cell surface markers. Different cell lines were used as controls for antibody staining for PSMA, CD45, and EpCAM, as follows: the C4-2 prostate cancer cells are PSMA+/EpCAM+/CD45− while the PC3 prostate cancer cells are PSMA−/EpCAM−/CD45−. The U937 leukemic cell line was used as a positive control for the leukocyte marker CD45. DAPI was used to stain the DNA. (TIFF) [file pone.0035976.s001.tiff]

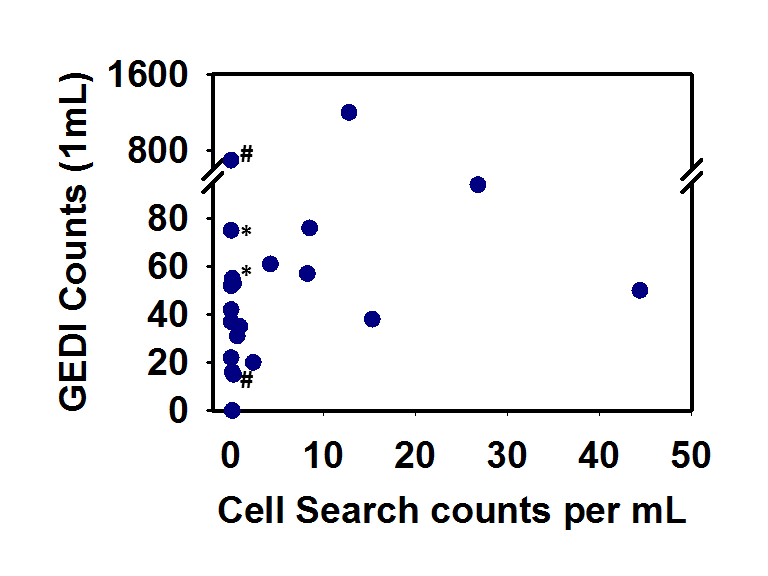

Supplement: Figure S2 — GEDI-CellSearch correlation. Correlation between the number of CTCs detected by the CellSearch® system vs. the GEDI system from same day blood draws. A correlation coefficient of r = 0.44 (outliers were removed with Cook's distance restriction) was determined. Hashtag and asterisk denote two pairs of data each taken on the same patient at two longitudinal time points. r is not changed significantly by inclusion or rejection of these points. (TIFF) [file pone.0035976.s002.tiff]

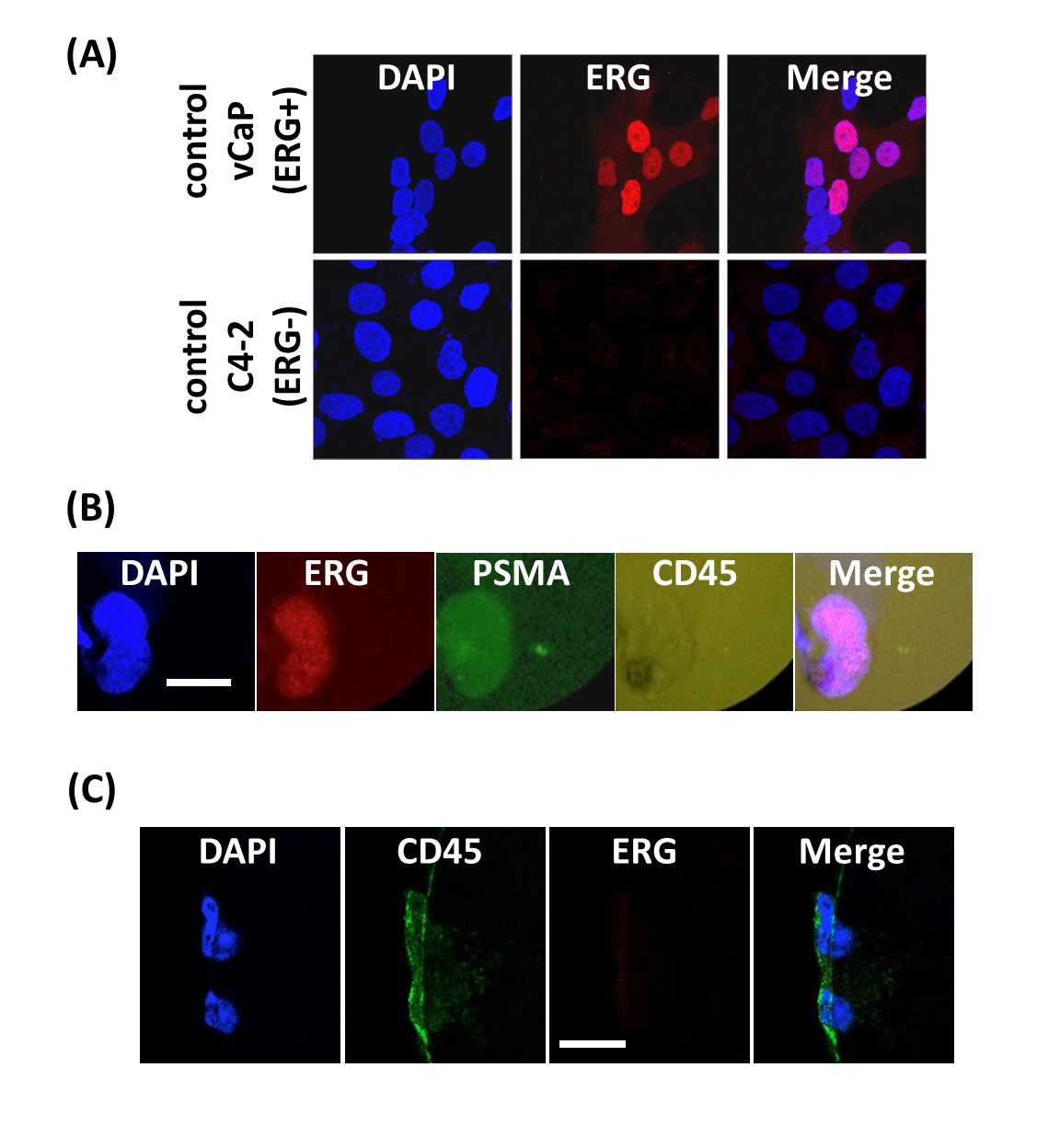

Supplement: Figure S3 — TMPRSS2:ERG detection by immunofluorescence on GEDI-captured cells. (A) The performance of the ERG antibody staining was tested in TMPRSS2:ERG fusion-positive (vCaP) and fusion-negative (C4-2) prostate cancer cell lines. Representative images acquired by confocal microscopy are displayed. Note the nuclear ERG staining in fusion-positive vCaP cells. (B) Two hundred vCaP cells were spiked in 1 ml of healthy-donor blood, flown through the GEDI device and processed for ERG immunofluorescence labeling. Nuclear ERG staining was detected in the GEDI-captured vCaP cells, identified as PSMA+/DAPI+/CD45− cells. (C) Representative example of ERG-negative/CD45+ leucocytes identified in the blood from a CRPC patient processed by the GEDI device as in Figure 4B. (TIFF) [file pone.0035976.s003.tiff]

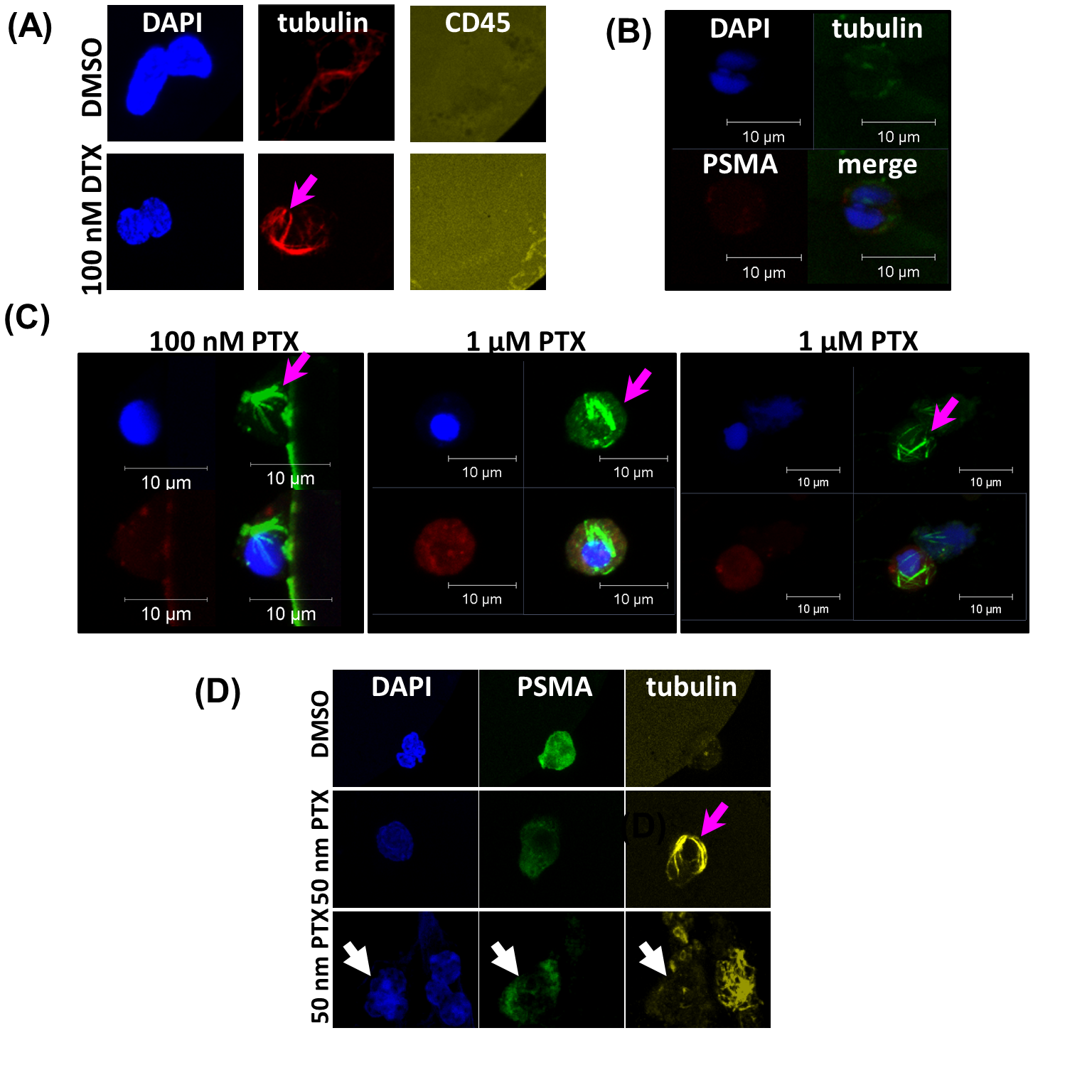

Supplement: Figure S4 — Additional examples of on-chip assessment of effective drug-target engagement from different CRPC patients: taxane-induced microtubule bundling and mitotic defects as evidence of drug-target engagement in GEDI-captured CTCs. (A) GEDI-captured CTCs from the same patient as in Figure 5B. PTX-induced prometaphase arrest of GEDI-captured CTCs provides additional evidence of effective drug-target engagement. (B) GEDI-captured CTCs from patient 3 following ex vivo on-chip treatment with 100 nM DTX do not show any evidence of microtubule response (bundling) to drug treatment. (C) GEDI-captured CTCs from patient 2 display microtubule bundling (arrow) following ex vivo on-chip treatment with 100 nM or 1 µM PTX. (D) GEDI-captured CTCs from patient 4 following ex vivo on-chip treatment with 50 nM PTX show heterogeneous response to drug treatment. Note, distinct microtubule bundling in a PSMA+ CTC (middle panel, barbed arrow) and no detectable microtubule network in another PSMA+ CTC from the same patient (bottom panel, standard arrow). The adjacent leucocyte (PSMA−) shows clear microtubule bundling in response to PTX treatment. (TIFF) [file pone.0035976.s004.tiff]

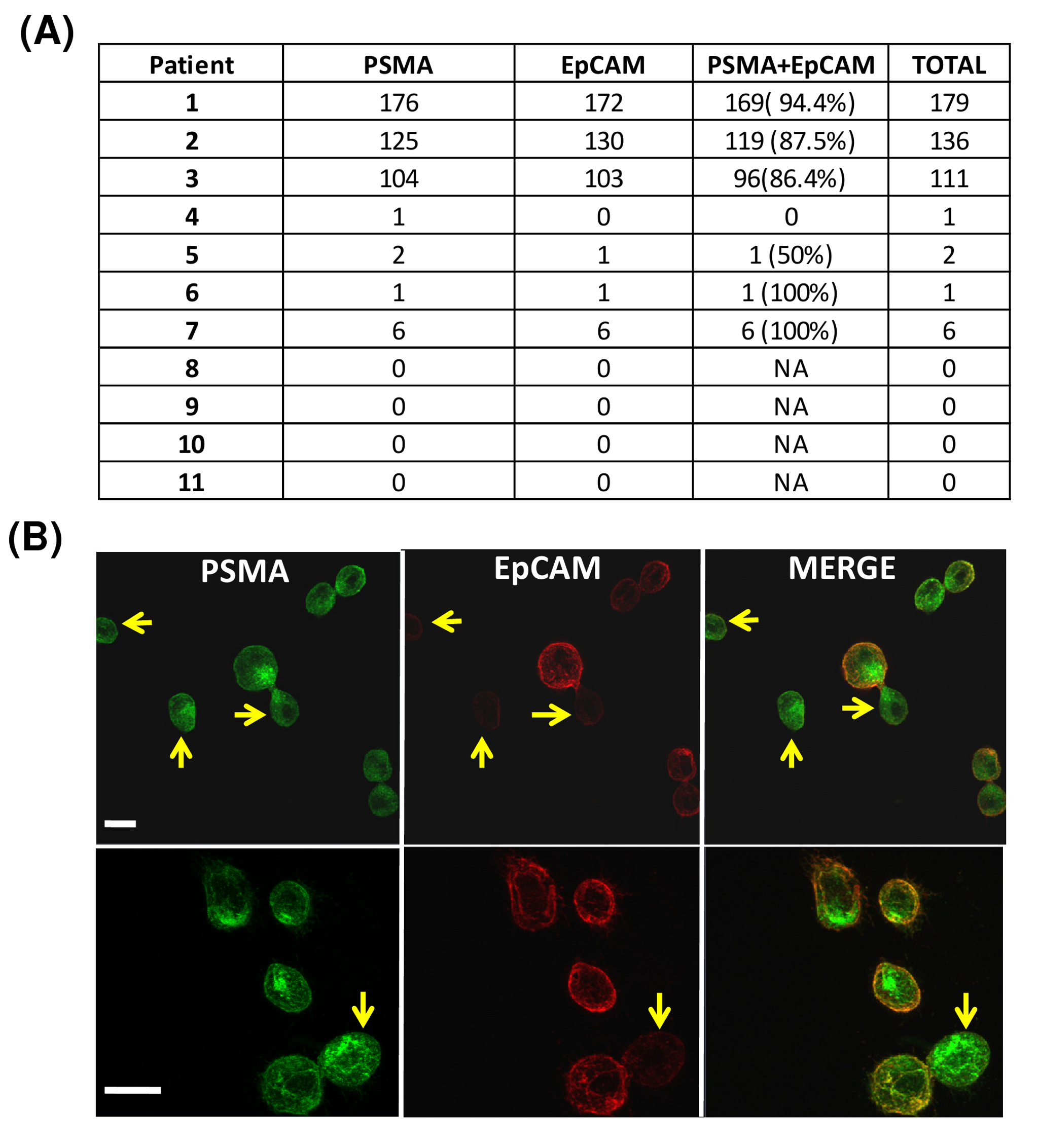

Supplement: Figure S5 — PSMA and EpCAM expression in CRPC patient CTCs isolated using CD45− immunodepletion. (A) Table showing the percentage of both PSMA and EpCAM positive CTCs from CRPC patients obtained using CD45-immunodepletion. (B) Representative images of CTCs isolated from 2 prostate cancer patients, stained for PSMA (Green) and EpCAM (Red) and analyzed by point scanning confocal microscopy. Scale Bar = 10 µm. The yellow arrows point to PSMA+/EpCAM dim staining. Notice the variability in EpCAM fluorescence intensity within each sample. (TIF) [file pone.0035976.s005.tif]
